# Improved ethanol productivity from lignocellulosic hydrolysates by *Escherichia coli* with regulated glucose utilization

**DOI:** 10.1186/s12934-018-0915-x

**Published:** 2018-05-02

**Authors:** Jinfeng Sun, Kangming Tian, Jie Wang, Zixing Dong, Xiaoguang Liu, Kugenthiren Permaul, Suren Singh, Bernard A. Prior, Zhengxiang Wang

**Affiliations:** 1 Center for Bioresource and Bioenergy, School of Biotechnology, Jiangnan University, 1800 Lihu Avenue, Wuxi, 214122 China; 2 Department of Biological Chemical Engineering, College of Chemical Engineering and Materials Science, Tianjin University of Science and Technology, TEDA, Tianjin, 300457 China; 3 School of Life Science and Food Engineering, Huaiyin Institute of Technology, 1st East Meicheng Road, Huaian, 223003 China; 4 Department of Biotechnology and Food Technology, Durban University of Technology, Durban, 4002 South Africa; 5 Department of Microbiology, University of Stellenbosch, Matieland, 7602 South Africa

**Keywords:** Ethanol, Thermo-regulated glucose utilization, Lignocellulosic hydrolysate, *Escherichia coli*

## Abstract

**Background:**

Lignocellulosic ethanol could offer a sustainable source to meet the increasing worldwide demand for fuel. However, efficient and simultaneous metabolism of all types of sugars in lignocellulosic hydrolysates by ethanol-producing strains is still a challenge.

**Results:**

An engineered strain *Escherichia coli* B0013-2021HPA with regulated glucose utilization, which could use all monosaccharides in lignocellulosic hydrolysates except glucose for cell growth and glucose for ethanol production, was constructed. In *E. coli* B0013-2021HPA, *pta*-*ackA*, *ldhA* and *pflB* were deleted to block the formation of acetate, lactate and formate and additional three mutations at *glk*, *ptsG* and *manZ* generated to block the glucose uptake and catabolism, followed by the replacement of the wild-type *frdA* locus with the *ptsG* expression cassette under the control of the temperature-inducible λ pR and pL promoters, and the final introduction of pEtac-PA carrying *Zymomonas mobilis pdc* and *adhB* for the ethanol pathway. B0013-2021HPA was able to utilize almost all xylose, galactose and arabinose but not glucose for cell propagation at 34 °C and converted all sugars to ethanol at 42 °C under oxygen-limited fermentation conditions.

**Conclusions:**

Engineered *E. coli* strain with regulated glucose utilization showed efficient metabolism of mixed sugars in lignocellulosic hydrolysates and thus higher productivity of ethanol production.

**Electronic supplementary material:**

The online version of this article (10.1186/s12934-018-0915-x) contains supplementary material, which is available to authorized users.

## Background

Lignocellulosic biomass holds tremendous potential for sustainable ethanol production to meet the increasing worldwide demand for ethanol, which is currently produced from starch- and sugar-based foodstuff materials [1]. Lignocellulose is composed of various hexose and pentose sugars, such as glucose and xylose [2, 3]. Economic feasibility of ethanol production from biomass, however, remains a challenge, though significant progress has been made in bioconversion of lignocellulose biomass to ethanol since 1970s, including: (1) pretreatment techniques of biomass, (2) enzymes for efficient saccharification, (3) new strains for metabolizing all or most of monosaccharides in lignocellulose hydrolysates, and (4) novel integrated processes for ethanol production and recovery.

Of all the components of lignocellulose-based ethanol production considered, strain development is still one of the most crucial elements for practical commercial process [4]. No single natural microorganism is known to be capable of efficiently converting all sugars from lignocellulose to ethanol [5, 6]. Although engineered ethanologenic *Escherichia coli* strains have the ability to metabolize various sugars from lignocellulosic biomass, their xylose utilization lags far behind that of glucose due to the preferential use of glucose as carbon and energy source by *E. coli*, a physiological phenomenon known as carbon catabolite repression (CCR) [7, 8]. Thus, a great deal of effort has been devoted to developing a single microorganism that can consume xylose and glucose simultaneously [9–14]. For example, to eliminate the CCR effect, strategies to disrupt CCR by inactivating phosphoenolpyruvate:glucose phosphotransferase system (PTS) components have been explored by various researchers [15–17].

In the present study, a metabolically engineered *E. coli* strain with regulated glucose utilization, which uses all monosaccharides from lignocellulose except glucose for cell propagation and all sugars for ethanol production, was constructed. The newly developed strain could utilize xylose, galactose and arabinose but glucose for cell duplication and its glucose catabolism pathway could be re-activated through switching-on transcription of *ptsG* at elevated temperature after cell duplication completed. A novel bioprocess for ethanol production from biomass was developed. This could provide an alternative route to highly efficient bioconversion of all sugars from biomass hydrolysates to ethanol.

## Methods

### Strains and plasmids

Strains and plasmids used in this study are listed in Table 1. Primers used in this study are listed in Additional file 1: Table S1. Cultures were stored at − 70 °C in 15% glycerol in the Culture and Information Center of Industrial Microorganism of China Universities at Jiangnan University (CICIM-CU, http://CICIM-CU.jiangnan.edu.cn). Unless otherwise stated, standard molecular biology protocols [18] were used for DNA manipulation.

**Table 1:** Strains and plasmids used in this study

Strains or plasmids	Genotype/relevant characteristics	Source or references
Strains		
*E. coli* B0013	Wild isolate	[20]
*E. coli* B0013-1030	B0013, Δ*pta*-*ackA*::*dif*,Δ*ldhA*::*dif*, Δ*pflB*::*dif*	[19]
*E. coli* B0013-1030H	B0013-1030, xylH*^a^	[20]
*E. coli* B0013-1031H	B0013-1030H, Δ*frdA*::*dif*	This study
*E. coli* B0013-1031HPA	B0013-1031H, pEtac-PA	This study
*E. coli* B0013-2020H	B0013-1030H, ∆*ptsG*::*dif*, ∆*glk*::*dif*, ∆*manZ*::*dif*	This study
*E. coli* B0013-2021H	B0013-2020H ∆*frdA*::kan-cI^ts^^857^-pR–pL-*ptsG*	This study
*E. coli* B0013-2021HPA	B0013-2021H, pEtac-PA	This study
Plasmid		
pMD19-T	*bla*; TA cloning vector	TaKaRa
pPL-kan	*bla*, *kan*, λcI^ts857^, pR pL	[24]
pT-kan-cI^ts857^-pR–pL-*ptsG*	*bla*, *kan*, *frdA*::kan-cI^ts857^-pR–pL	This study
pT-*frdA’*::kan-cI^ts857^-pR–pL-*ptsG*	*bla*, *kan*, *frdA*::kan-cI^ts857^-pR–pL-*ptsG*	This study
pEtac-PA	*kan*, *pdc* and *adhB* from *Zymomonas mobilis*	[13]

^a^ xylH* represents reversely mutated *xylH* ([20])

*Escherichia coli* B0013-1030 [19] was used as parent strain, in which *xylH*, encoding membrane component of high affinity xylose transporter [3], is naturally mutated [20]. Its XylH function was restored by homologous replacement of *xylH* to obtain *E. coli* B0013-1030H [20]. The *ptsG* coding for the enzyme IICB^Glc^ of the phosphoenolpyruvate:glucose phosphotransferase system for carbohydrate transport, *manZ* coding for the IID^Man^ domain of the mannose PTS permease, and *glk* coding for glucokinase [16, 21, 22] were disrupted in B0013-1030H to create the glucose-nonutilizing strain *E. coli* B0013-2020H according to the method described previously [23].

Integration of *ptsG* expression cassette under control of the temperature-inducible λ pR and pL promoters into the *frdA* of B0013-2020H to create B0013-2021H was performed according to the method described previously [24]. Briefly, fragment kan-cI^ts857^-pR–pL from plasmid pPL-kan was spliced with *ptsG* amplified from the chromosomal DNA of B0013 and cloned into pMD19T-vector to construct pT-kan-cI^ts857^-pR–pL-*ptsG*, in which λ pR and pL promoters was located before start codon of *ptsG*. The recombinant plasmid was digested with *Eco*RV and ligated with the fragment containing upstream and downstream homologous arms of *frdA* to obtain recombinant plasmid pT-*frdA’*::kan-cI^ts857^-pR–pL-*ptsG*. The cassette *frdA’*::kan-cI^ts857^-pR–pL-*ptsG* was amplified using primers *frdA*-p1 and *frdA*-p2, purified and integrated into the locus of *frdA* in the chromosome of B0013-2020H by electroporation [24] and *frdA* was simultaneously disrupted. Kanamycin resistant colonies were then selected on plates with 50 μg/ml of kanamycin. Integration of the cassette into *frdA* was confirmed by colony PCR (1785-bp for the wild-type *frdA* and 4034-bp after inactivation due to insertion of *ptsG* with λ pR and pL promoters) and by nucleotide sequencing. The resulting recombinant strain was designated B0013-2021H. The ethanol pathway encoded by pEtac-PA carrying *Z. mobilis pdc* and *adhB* [11] was transformed into B0013-2021H to develop ethanologenic recombinant B0013-2021HPA. As a control, B0013-1031HPA was constructed by disrupting *frdA* in B0013-1030H [24] and by subsequent introduction of pEtac-PA.

### Media

Luria–Bertani medium (LB) (5 g/l yeast extract, 10 g/l tryptone, and 5 g/l NaCl) is used for activation and cultivation of strains. Modified M9 medium [24] supplemented with 5 g/l of glucose or xylose was used for strain selection. As for solid medium, agar (15 g/l) is added. Modified M9 medium supplemented with 5 g/l of xylose and 50 g/l of glucose was used for shaking flask fermentation. Glucose and xylose were sterilized separately by autoclaving at 115 °C for 20 min. When it was necessary 100 μg/ml of ampicillin, 30 μg/ml of gentamycin or 50 μg/ml of kanamycin was added into the media.

### Fermentation experiments

Shaking flask fermentation was performed in 500-ml flasks containing 100 ml of medium. To prepare inoculum, one single colony from a fresh LB plate was transferred into 50 ml of LB medium in 250-ml flasks and then cultivated at 37 °C, 200 rpm for 10–12 h to an OD_600_ of 2.0–2.5. Cells were harvested by centrifugation (4300*g*, 10 min), washed and re-suspended with M9 medium, and then inoculated into 100 ml of fermentation medium to initial cell density of 0.1 at OD_600_. Fermentation was conducted using a “two-phase-two-temperature” strategy (aerobic cell growth at 34 °C, 200 rpm and then static oxygen-limited fermentation at 42 °C). The flask cultures were first cultivated in shaker at 34 °C, 200 rpm for 12 h and then transferred to static incubation for ethanol fermentation at 42 °C.

Ethanol fermentation experiments in a 7-l bioreactor (Bioflo110; New Brunswick Scientific Co., Inc., Edison, NJ) were accomplished with an initial 3 l working volume. Cells were cultured and collected as mentioned above and inoculated into 250-ml flasks containing 50 ml of modified M9 medium with 5 g/l of xylose to an initial cell density of 0.1 (OD_600_) and cultivated at 37 °C. Cultures were inoculated into 3 l of modified M9 medium containing 300 ml of corncob hydrolysate syrup. Using “two-phase-two-temperature” strategy as mentioned above, cell growth phase was carried out at 34 °C under aerobic conditions with agitation at 200–1000 rpm and sparging sterile air continuously at a rate of 0.1–1 vvm. When the cell mass reached OD_600_ of approximately 20, the incubation temperature was raised to 42 °C and maintained for 45 min. And then the oxygen limited fermentation was initiated by stopping air flow and reducing agitation speed to 100 rpm and additional 300 ml of corncob hydrolysate was added at this moment. The same fermentation was carried out with B0013-1031HPA as control except that incubation temperature for cell growth and fermentation was both at 37 °C. The pH was maintained at 7.0 by automatically feeding concentrated NH_4_OH or 10% H_2_SO_4_ (v/v) during the fermentation. The corncob hydrolysate syrup used in this study was prepared based on 70% (w/w) concentrated xylose crystallization mother liquid (Futian Pharmaceutical Company Ltd., China) supplemented with 364 g/l glucose with the volume ratio 1:1. The final syrup contained 266 g/l of glucose, 200 g/l of xylose, 114.5 g/l of arabinose and 27 g/l of galactose.

### Analysis of relative expression levels of *ptsG* using real-time quantitative PCR

Cells grown in LB medium at 34 °C overnight were collected and washed 3 times with cold M9 medium. The cells were then resuspended in cold modified M9 medium containing 5 g/l glucose to OD_600_ of 0.1 and then incubated for 2 h at 34 or 42 °C. The cells were recovered and total RNA was extracted using the ChargeSwitch^®^ Total RNA Cell Kits (Invitrogen) according to the protocol with on-column DNase digestion. RNA was eluted with Elution Buffer to a sterile microcentrifuge tube. cDNA synthesis was performed using 3 μg of total RNA with PrimeScript Reverse Transcriptase (TaKaRa, Dalian, China) in a 20 μl reaction volume according to the manufacturer’s instructions. Real-time quantitative PCR was performed on Step One System (ABI, USA) to determine mRNA level of *ptsG*. The PCR reaction system (20 μl) and conditions were according to the method as described [25]. The Ct values were used to quantify the relative expression levels of *ptsG* by the 2^−ΔCt^ method with the constitutively expressed *gapA*, encoding glyceraldehyde-3-phosphate dehydrogenase A, as the internal control [26].

### Preparation of cell extract and activity assay of phosphoenolpyruvate:glucose phosphotransferase (PTase), pyruvate decarboxylase (PDC) and alcohol dehydrogenase II (ADH II)

Cells grown in LB medium at 34 °C overnight were collected and washed 3 times with sodium chloride solution (0.85%, w/v). The cells were then resuspended in modified M9 medium containing 5 g/l glucose and incubated for 2 h at 34 or 42 °C. The cells were recovered for enzyme activity assay. The cell lysates were prepared according to the method as described previously with some modification [27]. Briefly, about 0.2 g of wet cells were washed with ice-cold sodium chloride solution (0.85%, w/v) and resuspended in 2 ml of 50 mM morpholinepropane-sulfonic acid (MOPS; pH 7.5) buffer containing 10% (v/v) glycerol, 5 mM EDTA, 1 mM benzamidine and 2 mM dithiothreitol. After passage through a French Press Cell (Aminco, USA) and incubation with RNase and DNase for 1 h at room temperature, the solution was centrifuged at 27,000×*g* and 4 °C for 30 min, and the supernatant was desalted by passing through a PD-10 column (Amersham, GE-Beijing, China).

PTase activity was measured in 50 mM MOPS buffer (pH 7.5) containing 5 mM MgCl_2_, 10 mM NADH (Sangon Biotech, Shanghai, China) and 100 μg cell extract at 25 °C. The reaction was started by addition of 5 mM glucose, 5 mM phosphoenolpyruvate and 10 U lactate dehydrogenase (Sangon Biotech, Shanghai, China). The concentration of NADH was measured at 340 nm. One unit of PTase activity was defined as the quantity of enzyme required to transform 1 μmol of NADH to NAD^+^ per minute. PDC and ADH II activities were measured according to the procedure as described previously [13, 28]. Protein concentrations were determined by the method of Bradford with crystalline bovine serum albumin fraction V (Sangon Biotech, Shanghai, China) as described [13, 24].

### Analytical methods

Sampling was conducted periodically. Glucose, xylose, arabinose, galactose and ethanol were measured by HPLC according to the method described previously [23] using a HPLC system equipped with Dionex p680 pump (Dionex Corporation, Sunnyvale, CA), a Shodex SH-1011 column (Shodex SH-1011 H610009; Showa Denko K.K., Kawasaki, Japan), and a refractive index detector. The samples were run at 60 °C and eluted at 1.0 ml/min with 0.01 M sulfuric acid. Cell density was monitored turbidimetrically at 600 nm (1 cm light path) using a UNICO UV2000 spectrophotometer. Dry cells weight was calculated using a standard curve (1 OD_600_ = 0.38 g/l DCW) [24].

## Results and discussion

### Construction of regulated glucose-utilizing strain *E. coli* B0013-2021H

**Fig. 1:**
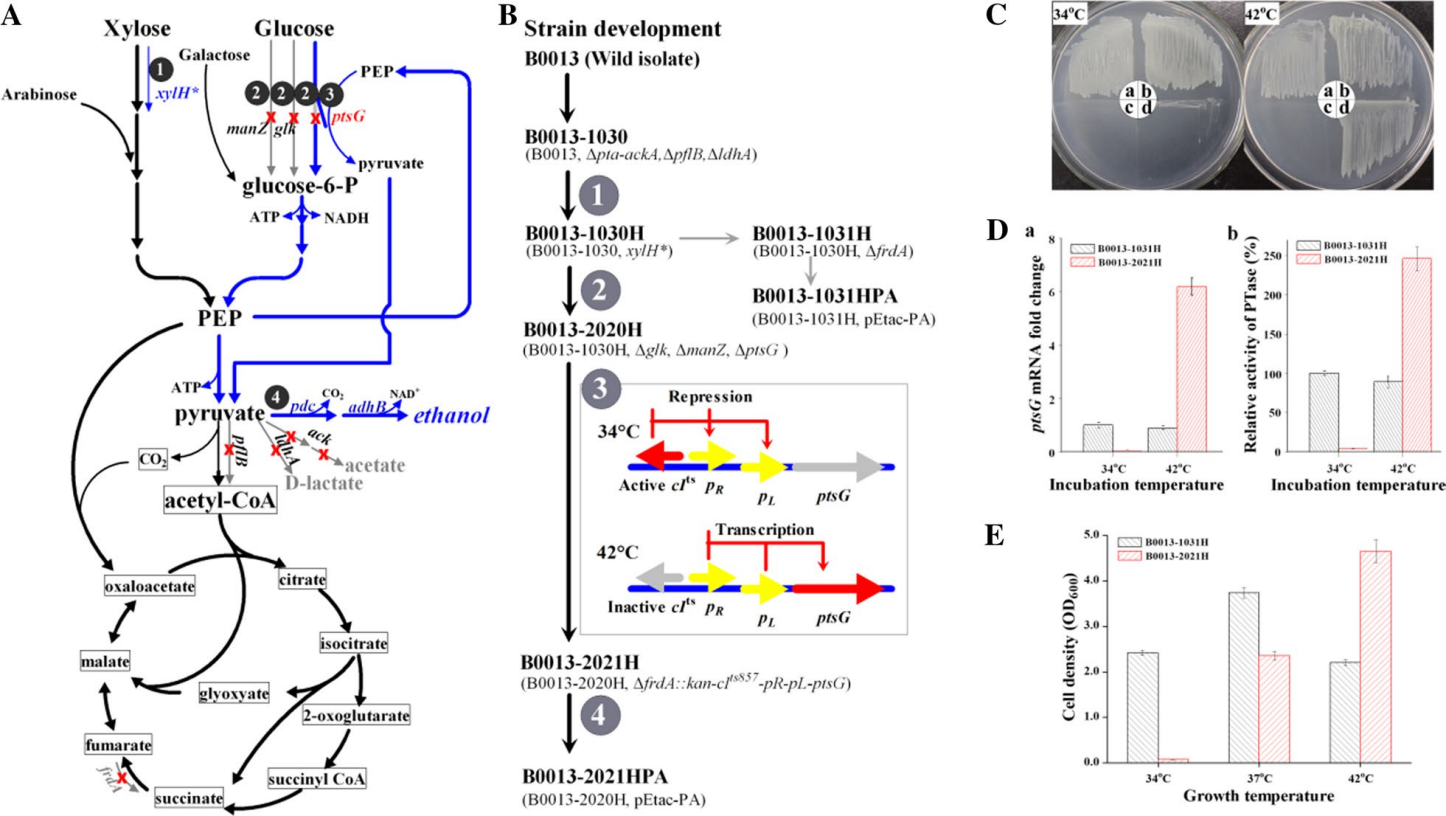
Construction and characterization of regulated glucose-utilizing *E. coli* B0013-2021H. **A** Modified metabolic pathways of *E. coli* B0013 and its derivatives; **B** development flowchart of *E. coli* B0013-2021H and relatives; **C** growth characterization of *E. coli* B0013-2021H on glucose at different temperatures. The cells were cultivated on M9 plates with 5 g/l of glucose at 34 or 42 °C. a: *E. coli* B0013; b: B0013-1031H; c: B0013-2020H; d: B0013-2021H; **D** (a) *ptsG* mRNA fold change quantified by real-time quantitative PCR; (b) Expression level of phosphoenolpyruvate:glucose phosphotransferase in *E. coli* B0013-2021H at different incubation temperatures; **E** growth characterization of *E. coli* B0013-2021H and B0013-1031H. The cells grew in M9 medium supplemented with 5 g/l of glucose at 34, 37 or 42 °C and 200 rpm for 12 h. The cell densities were determined at 600 nm

To enable controllable glucose utilization by *E. coli*, glucose-nonutilizing strain *E. coli* B0013-2020H was successfully constructed by sequentially disrupting *ptsG*, *manZ* and *glk*, which were previously identified to be essential for glucose uptake and metabolism in *E. coli* [16, 17, 21, 22] (Fig. 1). B0013-2020H failed to grow on glucose (Fig. 1c). Growth characteristics of *E. coli* B0013-2020H on glucose were also examined by cultivation in M9 medium containing xylose or glucose in shaking flasks at 37 °C and 200 rpm. After cultivation for 12 h, B0013-2020H proliferated to cell density of 3.78 on xylose but failed to grow on glucose (data not shown), indicating that its glucose metabolizing pathway was blocked.

To restore and artificially regulate glucose utilization of B0013-2020H, a new strain, designated B0013-2021H, was developed in which *ptsG* was expressed under the control of λ pR and pL promoters, a temperature-inducible switch [24] (Fig. 1b). Growth characteristics of B0013-2021H, B0013-2020H, and the parental strains B0013 and B0013-1031H were compared by cultivating on M9 plates supplemented with glucose at 34 or 42 °C. Both B0013-2021H and B0013-2020H could not grow on glucose at 34 °C, while B0013-2021H, B0013 and B0013-1031H showed robust growth on glucose at 42 °C except B0013-2020H (Fig. 1c). Subsequently, real-time quantitative PCR was employed to determine the mRNA levels of *ptsG* at 34 or 42 °C. The transcription of *ptsG* in B0013-2021H was highly induced at 42 °C with more than sixfolds of that in B0013-1031H (Fig. 1Da). The PTase activities in B0013-2021H and B0013-1031H incubated at 34 or 42 °C were further determined. B0013-2021H exhibited trace amounts of PTase activity at 34 °C and about threefolds of higher PTase activity at 42 °C in comparison to that of B0013-1031H (Fig. 1Db), illustrating that the expression of *ptsG* in B0013-2021H is fully controlled by λ pR–pL promoter.

Growth characteristics of B0013-2021H were further investigated by cultivation in M9 medium with 5 g/l of glucose at 34, 37 or 42 °C and 200 rpm (Fig. 1e). Cell density for B0013-2021H remained unchanged after incubation on glucose for 12 h at 34 °C because *ptsG* expression was not switched on (Fig. 1e). At 37 °C, B0013-2021H partially restored its growth on glucose with an increase in cell density to OD_600_ of 2.34, slightly slower growth than that of B0013-1031H (Fig. 1e). When incubation temperature was elevated to 42 °C, B0013-2021H grew more vigorously than B0013-1031H due to the full operation of temperature switch of *ptsG* at 42 °C (Fig. 1e). The results further confirmed that the expression of *ptsG* in B0013-2021H was strictly under the control of the temperature-inducible tandem λ pL and pR promoters and elevated incubation temperature switched on the transcription of *ptsG* in B0013-2021H.

Preferential use of glucose is a major factor limiting economical lignocellulose-based ethanol production. *E. coli* B0013-2021H with controllable metabolism of glucose provides a simple solution to achieving efficient metabolism of all sugars from lignocellulosic biomass: under aerobic conditions, a relatively low temperature (34 °C) was adopted so that glucose utilization was switched off and the strain utilized all sugars except glucose to grow and the inhibition effect of glucose on other sugars’ utilization was relieved. When the process was shifted to anaerobic conditions, the temperature switch was activated at elevated temperature (42 °C) and thus the expression of *ptsG*, leading to glucose uptake and catabolism, and efficient production of ethanol by fermenting glucose and other sugars in biomass hydrolysates.

### B0013-2021HPA utilized xylose for growth and glucose for ethanol fermentation in shaking flasks

The ethanol synthesis pathway encoded by pEtac-PA was finally introduced into B0013-2021H, resulting in ethanologenic strain B0013-2021HPA (Fig. 1b). As a control, B0013-1031HPA was constructed by deleting *frdA* in B0013-1030H and the introduction of pEtac-PA (Fig. 1b). At 34 and 42 °C, the activities of PDC in B0013-2021HPA were determined to be 6.8 ± 1.22 U/mg protein and 6.7 ± 1.35 U/mg protein of PDC, respectively, while the activities of ADH II were 3.1 ± 0.65 U/mg protein and 3.2 ± 0.57 U/mg protein, respectively.

**Fig. 2:**
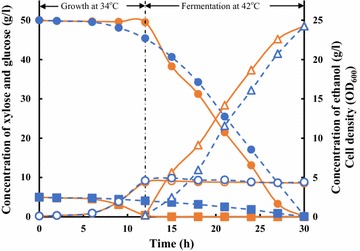
Ethanol fermentation by *E. coli* B0013-2021HPA. In 500-ml shaking flasks containing 100 ml of M9 medium with 5 g/l of xylose and 50 g/l of glucose, the cultures were cultivated at 34 °C and 200 rpm during aerobic phase and ethanol fermentation was carried out statically at 42 °C using a “two-phase-two-temperature” strategy. Solid line: B0013-2021HPA; dotted line: B0013-1031HPA. Solid square: xylose; solid circle: glucose; triangle: ethanol; circle: cell mass

To evaluate fermentation performance of B0013-2021HPA, ethanol fermentation was conducted in shaking flasks using a “two-phase-two-temperature” strategy, as described in “Methods”, in M9 medium supplemented with 5 g/l of xylose and 50 g/l of glucose. B0013-2021HPA utilized all of the xylose for growth under aerobic condition at 34 °C whereas glucose concentration remained approximately constant at 50 g/l (Fig. 2). B0013-2021HPA grew to a cell density of 4.9 (OD_600_) at 12 h and 34 °C and the cultures were then incubated at 42 °C for ethanol fermentation. After 18 h of fermentation, 50 g/l of glucose was exhausted and converted to 24.3 g/l of ethanol (Fig. 2) with the yield of 48.4 g/100 g glucose. Under the same conditions, *E. coli* B0013-1031HPA mainly consumed glucose for cell growth during the aerobic phase and converted residue glucose and xylose to ethanol during fermentation phase, with the similar yield as that of B0013-2021HPA. Consumption rate of glucose of B0013-1031HPA [2.51 g/(l·h)] was less than that of B0013-2021HPA [3.08 g/(l·h)] because *ptsG*, under the control of λ pR and pL promoters, in B0013-2021HPA was activated and highly expressed.

### *E. coli* B0013-2021HPA converted mixed sugars to ethanol in bioreactor

**Fig. 3:**
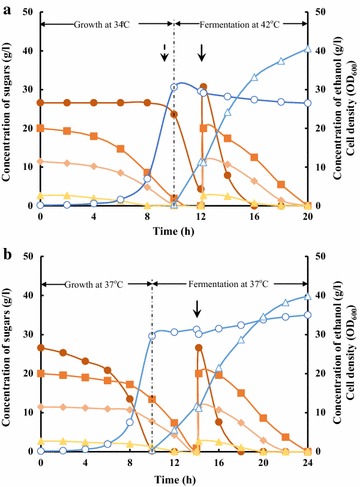
Ethanol fermentation of *E. coli* B0013-2021HPA from corncob hydrolysate in a 7-l bioreactor. Pre-cultured cells of B0013-2021HPA (**a**) were inoculated into 3 l of modified M9 medium supplemented with 300 ml of corncob hydrolysate. Using a “two-phase-two-temperature” strategy as mentioned in “Methods”, cell growth phase was controlled at 34 °C under aerobic conditions and ethanol fermentation was conducted at 42 °C under oxygen-limited conditions. As for B0013-1031HPA (**b**), temperature was set at 37 °C, its optimal temperature, for the two phases. Solid circle: glucose; solid triangle: galactose; solid diamond: arabinose; solid square: xylose; triangle: ethanol; circle: cell mass; dotted arrow: temperature switching point (from 34 to 42 °C), 45 min ahead of transition to oxygen-limited condition; arrow: time point of addition of 300 ml of the corncob hydrolysate

Ethanol fermentation performance of B0013-2021HPA on mixed sugars, from corncob hydrolysate for example, was further verified in a 7-l bioreactor with an initial 3 l working volume containing 300 ml of corncob hydrolysate for cell growth and additional 300 ml of corncob hydrolysate was supplemented for ethanol production. The results are summarized in Fig. 3 and Table 2. Significantly, B0013-2021HPA utilized xylose, galactose and arabinose for growth, while glucose was not consumed at all during the aerobic growth at 34 °C and utilization of glucose was initiated after temperature switch to 42 °C. After 12 h of fermentation at 42 °C, the total amount of 260.9 g mixed sugars, including residual glucose and xylose as well as mixed sugars from added corncob hydrolysate were converted to 127.7 g ethanol with the productivity of 4.06 g/(l·h) and sugar consumption rate of 8.28 g/(l·h) (Fig. 3a, Table 2). All these performances are significantly improved in comparison to those of B0013-1031HPA, whose ethanol productivity was 2.85 g/(l·h) and sugar consumption rate was 5.81 g/(l·h) (Fig. 3b, Table 2).

**Table 2:** Consumption rates of sugars and ethanol productivity by B0013-2021HPA and B0013-1031HPA in a 7-l bioreactor

Strains	Consumption rate of sugars^a^ [g/(l·h)]			Productivity of ethanol^b^ [g/(l·h)]
	Whole process	Cell growth	Fermentation	
B0013-2021HPA	5.90	3.52	8.28	4.06
B0013-1031HPA	4.74	3.67	5.81	2.85

^a^ Consumption rate of sugars was calculated based on the sugars consumed during aerobic phase or fermentation phase

^b^ Productivity of ethanol was calculated based on the time during fermentation phase

Lignocellulosic hydrolysate, such as corncob hydrolysate, comprises a mixture of sugars, mainly glucose and xylose [29, 30]. In *E. coli* and many other microorganisms, glucose is preferentially utilized and other sugars would be used only when glucose is nearly depleted due to CCR [8, 15]. To realize simultaneous utilization of pentose and hexose, previous work focused on development of engineered strains, including construction of catabolite repression mutant by mutation of *ptsG* to achieve simultaneous fermentation of mixed glucose, xylose and arabinose to ethanol with the yield of 0.45 g/g, the productivity of 0.75 g/(l·h) and sugar consumption rate of 1.31 g/(l·h) in 70 h [15]. Also, simultaneous consumption of mixed sugars was achieved by strain adaptation through substrate-selective inoculum preparation or late addition of saccharifying enzyme to delay releasing of glucose, resulting in maximum ethanol productivity of 0.72 g/(l·h) in 40 h and 0.78 g/(l·h) in 48 h, respectively, from corn stover hydrolysate by *E. coli* FBR5 [31].

Aerobic condition is considered to be more beneficial to utilization of xylose in *E. coli* [32, 33]. In this work, the metabolic inefficiency for ethanol production from mixed sugars in lignocellulosic hydrolysates was alternatively solved by switching off glucose utilization to allow growth of cells on xylose and other sugars but glucose during the aerobic cell growth phase and by switching on glucose utilization to enable efficient conversion of glucose and other sugars to ethanol.

Notably, sugars consumption patterns of our developed strain were different from other reports [15, 31, 34–36]. This may probably due to the differences in genetic background among *E. coli* strains.

## Conclusions

An alternative bioprocess for ethanol production from biomass was successfully developed using a strain genetically engineered to turn on or off glucose utilization in *E. coli* by a temperature switch, thus relieving the inhibition effect of glucose on utilization of other sugars. The glucose-utilization switched-off cells can preferentially utilize xylose and other sugars present in lignocellulosic hydrolysates for cell growth, while glucose-utilization switched-on cells can efficiently convert glucose and other sugars to ethanol during anaerobic stage for ethanol fermentation. Significantly, utilization efficiency of sugars in lignocellulosic hydrolysates was improved and so was the economic feasibility of ethanol production from biomass.

## Additional file

**Additional file 1: Table S1.** Primers used in this study.

## References

[1] Banerjee S, Mudliar S, Sen R, Giri B, Satpute D, Chakrabarti T, Pandey R (2010). Commercializing lignocellulosic bioethanol: technology bottlenecks and possible remedies. Biofuel Bioprod Biorefining.

[2] Rubin EM (2008). Genomics of cellulosic biofuels. Nature.

[3] Khankal R, Chin JW, Cirino PC (2008). Role of xylose transporters in xylitol production from engineered *Escherichia coli*. J Biotechnol.

[4] Stephanopoulos G (2007). Challenges in engineering microbes for biofuels production. Science.

[5] Garcia Sanchez R, Karhumaa K, Fonseca C, Sànchez Nogué V, Almeida JRM, Larsson CU, Bengtsson O, Bettiga M, Hahn-Hägerdal B, Gorwa-Grauslund MF (2010). Improved xylose and arabinose utilization by an industrial recombinant *Saccharomyces cerevisiae* strain using evolutionary engineering. Biotechnol Biofuels.

[6] Dellomonaco C, Fava F, Gonzalez R (2010). The path to next generation biofuels: successes and challenges in the era of synthetic biology. Microb Cell Fact.

[7] Yomano LP, York SW, Shanmugam KT, Ingram LO (2009). Deletion of methylglyoxal synthase gene (*mgsA*) increased sugar co-metabolism in ethanol-producing *Escherichia coli*. Biotechnol Lett.

[8] Karimova G, Ladant D, Ullmann A (2004). Relief of catabolite repression in a cAMP-independent catabolite gene activator mutant of *Escherichia coli*. Res Microbiol.

[9] Yomano LP, York SW, Zhou S, Shanmugam KT, Ingram LO (2008). Re-engineering *Escherichia coli* for ethanol production. Biotechnol Lett.

[10] Dien BS, Nichols NN, O’Bryan PJ, Bothast RJ (2000). Development of new ethanologenic *Escherichia coli* strains for fermentation of lignocellulosic biomass. Appl Biochem Biotechnol.

[11] Zhang M, Eddy C, Deanda K, Finkelstein M, Picataggio S (1995). Metabolic engineering of a pentose metabolism pathway in ethanologenic *Zymomonas mobilis*. Science.

[12] Karhumaa K, Garcia Sanchez R, Hahn-Hägerdal B, Gorwa-Grauslund MF (2007). Comparison of the xylose reductase-xylitol dehydrogenase and the xylose isomerase pathways for xylose fermentation by recombinant *Saccharomyces cerevisiae*. Microb Cell Fact.

[13] Sun JF, Xu M, Zhang F, Wang ZX (2004). Novel recombinant *Escherichia coli* producing ethanol from glucose and xylose. Acta Microbiol Sin.

[14] van Maris AJ, Winkler AA, Kuyper M (2007). Development of efficient xylose fermentation in *Saccharomyces cerevisiae*: xylose isomerase as a key component. Adv Biochem Eng Biotechnol.

[15] Nichols NN, Dien BS, Bothast RJ (2001). Use of catabolite repression mutants for fermentation of sugar mixtures to ethanol. Appl Microbiol Biotechnol.

[16] Gosset G (2005). Improvement of *Escherichia coli* production strains by modification of the phosphoenolpyruvate:sugar phosphotransferase system. Microb Cell Fact.

[17] Hernández-Montalvo V, Valle F, Bolivar F, Gosset G (2001). Characterization of sugar mixtures utilization by an *Escherichia coli* mutant devoid of the phosphotransferase system. Appl Microbiol Biotechnol.

[18] Sambrook J, Russell DW (2001). Molecular cloning: a laboratory manual. 2001.

[19] Cao JL, Zhou L, Zhang L, Wang ZX, Shi GY (2010). Construction and fermentation of succinate-producing recombinant *Escherichia coli*. Chin J Appl Environ Biol.

[20] Sun JF, Tian KM, Shen W, Chen XZ, Wang ZX (2017). Genetic nature of xylose metabolism, differences between *Escherichia coli* strains. Chin J Food Ferment Ind.

[21] Huber F, Erni B (1996). Membrane topology of the mannose transporter of *Escherichia coli* K12. Eur J Biochem.

[22] Deutscher J, Francke C, Postma PW (2006). How phosphotransferase system-related protein phosphorylation regulates carbohydrate metabolism in bacteria. Microbiol Mol Biol Rev.

[23] Zhou L, Zuo ZR, Chen XZ, Niu DD, Tian KM, Prior BA, Shen W, Shi GY, Singh S, Wang ZX (2011). Evaluation of genetic manipulation strategies on d-lactate production by *Escherichia coli*. Curr Microbiol.

[24] Zhou L, Niu DD, Tian KM, Chen XZ, Prior BA, Shen W, Shi GY, Singh S, Wang ZX (2012). Genetically switched d-lactate production in *Escherichia coli*. Metab Eng.

[25] Wang RJ, Sui PC, Hou XJ, Cao T, Jia LG, Lu FP, Singh S, Wang ZX, Liu XG (2017). Cloning and identification of a novel steroid 11 α-hydroxylase gene from *Absidia coerulea*. J Steroid Biochem Mol Biol.

[26] Livak KJ, Schmittgen TD (2001). Analysis of relative gene expression data using real-time quantitative PCR and the 2^−ΔΔCt^ method. Methods.

[27] Wang ZX, Brämer C, Steinbüchel A (2003). Two phenotypically compensating isocitrate dehydrogenases in *Ralstonia eutropha*. FEMS Microbiol Lett.

[28] Ingram LO, Conway T (1988). Expression of different levels of ethanologenic enzymes from *Zymomonas mobilis* in recombinant strains of *Escherichia coli*. Appl Environ Microbiol.

[29] Andersen RL, Jensen KM, Mikkelsen MJ (2015). Continuous ethanol fermentation of pretreated lignocellulosic biomasses, waste biomasses, molasses and syrup using the anaerobic, thermophilic bacterium *Thermoanaerobacter italicus* Pentocrobe 411. PLoS ONE.

[30] Sheehan J, Himmel M (1999). Enzymes, energy, and the environment: a strategic perspective on the US Department of Energy’s research and development activities for bioethanol. Biotechnol Prog.

[31] Saha BC, Qureshi N, Kennedy GJ, Cotta MA (2015). Enhancement of xylose utilization from corn stover by a recombinant *Escherichia coli* strain for ethanol production. Bioresour Technol.

[32] Cirino PC, Chin JW, Ingram LO (2006). Engineering *Escherichia coli* for xylitol production from glucose–xylose mixtures. Biotechnol Bioeng.

[33] Hasona A, Kim Y, Healy FG, Ingram LO, Shanmugam KT (2004). Pyruvate formate lyase and acetate kinase are essential for anaerobic growth of *Escherichia coli* on xylose. J Bacteriol.

[34] Lindsay SE, Bothast RJ, Ingram LO (1995). Improved strains of recombinant *Escherichia coli* for ethanol production from sugar mixtures. Appl Microbiol Biotechnol.

[35] Dien BS, Hespell RB, Wyckoff HA, Bothast RJ (1998). Fermentation of hexose and pentose sugars using a novel ethanologenic *Escherichia coli* strain. Enzyme Microb Technol.

[36] Lee SK, Kim GH, Jeong SH, Kim SM, Choi BY. Method for preparing mutant *Escherichia coli* capable of simultaneously utilizing glucose and xylose. United States Patent, US9284582B2. 2016.

