# Supplementary material for: Improved ethanol productivity from lignocellulosic hydrolysates by Escherichia coli with regulated glucose utilization
Source: Microb Cell Fact. 2018 May 2;17:66. doi: 10.1186/s12934-018-0915-x (PMC5930954; doi:10.1186/s12934-018-0915-x)
Supplement: Supplementary file 1 — Additional file 1: Table S1. Primers used in this study. [file 12934_2018_915_MOESM1_ESM.docx]

Table S1 Primers used in this study

| Primers | Sequence (5′-3′) restriction sites are italic/underlined | Restriction sites |
| --- | --- | --- |
| ptsG1 | CCG*GAATTC*TCGGTAAATCGCTGATGCTGCC | *Eco*RI |
| ptsG2 | CCG*GAATTC*CGGGTAATACATGCGTCGAGGTTAG | *Eco*RI |
| YptsG1 | GTCAAAATGTGCAACTTCTCCAATG |  |
| glk-p1-up | CGGATGGAGCATGTTCTATTC |  |
| glk-p2-down | CATAAAGGTGTGAGCCAGGC |  |
| T-glk-p1-r | GGCAATTGAGAACGCCCAGGTATG |  |
| T-glk-p2-r | CGCCAGACCTTAGGTCACATTCTGTAA |  |
| Y-glk-p2 | TTACAGAATG TGACCTAAGG TCTGGCG |  |
| manZ-p1-up | ACCAGCGAAGTACAGAACATGC |  |
| manZ-p2-down | ACGCACCATGATTCGTGACGTTG |  |
| T-manZ-p1-r | AGTGCCTGCATACGTTCGAAGTTCCA |  |
| T-manZ-p2-r | GTCCAGACTATTCTGGACCAGTTA |  |
| Y-manZ-p2 | TAACTGGTCC AGAATAGTCT GGAC |  |
| cI857-p1 | TAA*GATATC*CCATGATTACGAATTGCCGGC | *Eco*RV |
| PL-ptsG-overlap2 | GCTCCTGAGTATGGGTGCTTTCAGTATCACCGCCAGTGGT |  |
| ptsG-p12 | AAGCACCCATACTCAGGAGC |  |
| ptsG-p22 | TAA*GATATC*TGGCTGCCTTAGTCTCC | *Eco*RV |
| frdA-p1 | CATTCGCCTTCTCCTTCTTA |  |
| frdA-p2 | ATCTTGCCATTGTAGGCG |  |
| Y-frdA'-p2 | TTCTTCCCGTATGCCCAA |  |
| Primers for real-time quantitative PCR | | |
| ptsG’1 | AAAGCACCCATACTCAGGAG |  |
| ptsG’2 | GGATACGCCATCGTTATTG |  |
| gapA’1 | TGGGACGAAGTTGGTGTT |  |
| gapA’2 | CGATGATGCCGAAGTTATC |  |
